# Fixed Dose Versus Loose Dose: Analgesic Combinations

**DOI:** 10.7759/cureus.33320

**Published:** 2023-01-03

**Authors:** Joseph Pergolizzi, Giustino Varrassi, Jo Ann K LeQuang, Frank Breve, Peter Magnusson

**Affiliations:** 1 Cardiology, Native Cardio, Inc., Naples, USA; 2 Research, Paolo Procacci Foundation, Roma, ITA; 3 Pain Management, NEMA Research, Inc., Naples, USA; 4 Pharmacy, Temple University, Philadelphia, USA; 5 School of Medicine, Örebro University, Örebro, SWE; 6 Cardiology, Center of Research and Development Region Gävleborg/Uppsala University, Gävle, SWE; 7 Medicine and Cardiology Research Unit, Karolinska Institutet, Stockholm, SWE

**Keywords:** pain, opioids, nonopioid analgesics, loose dose combination analgesics, fixed-dose combination analgesics, analgesia

## Abstract

Combinations of drugs may be fixed (two or more entities in a single product) or loose (two or more agents taken together but as individual agents) to help address multimechanistic pain. The use of opioids plus nonopioids can result in lower opioid consumption without sacrificing analgesic benefits. Drug combinations may offer additive or synergistic benefits. A variety of fixed-dose combination products are available on the market such as diclofenac plus thiocolchicoside, acetaminophen and caffeine, acetaminophen and opioid, ibuprofen and acetaminophen, tramadol and acetaminophen, and others. Fixed-dose combination products offer predictable pharmacokinetics and pharmacodynamics, known adverse events, and can reduce the pill burden. However, they are limited to certain drug combinations and doses; loose dosing allows prescribers the versatility to meet individual patient requirements as well as the ability to titrate as needed. Not all drug combinations offer synergistic benefits, which depend on the drugs and their doses. Certain drugs offer dual mechanisms of action in a single molecule, such as tapentadol, and these may further be used in combination with other analgesics. New technology allows for co-crystal productions of analgesic agents which may further improve drug characteristics, such as bioavailability. Combination analgesics are important additions to the analgesic armamentarium and may offer important benefits at lower doses than monotherapy.

## Introduction and background

Combination analgesic regimens are becoming increasingly important both to spare opioid consumption and to control multimechanistic or complex pain states. It was the pioneering work of R. W. Houde and colleagues who, in the 1960s, advocated for methodologies to evaluate analgesic efficacy in patients and the utility of analgesic combinations, particularly aspirin and morphine, among others [1]. Combinations of a nonopioid analgesic with a relatively small dose of opioids may allow a patient to achieve pain relief equivalent to opioid monotherapy but with a reduction in the patient’s cumulative exposure to opioids [2]. Nevertheless, combining analgesics in ways that are safe, effective, and beneficial to the patient requires careful prescribing considerations. In particular, fixed-dose versus loose-dose analgesic regimens offer particular challenges to clinicians in terms of terminology, isobolographic determinations of additive versus synergistic benefits, and the utility of specific multimechanistic agents. Polypharmacy may also play a role in overdose, particularly due to drug-drug interactions such as two agents that have a synergistic effect on respiratory depression.

There is a lack of consensus on the terminology used to describe combination analgesic regimens. Combination regimens are more than polypharmacy, they incorporate specific agents used purposefully together to achieve a specific analgesic objective. Furthermore, just because two or more agents can be safely combined does not mean that they should be combined; some combinations do not offer improved pain control. Other combinations may not be safe or may increase the risk or severity of adverse events. In this context, newer agents based on multiple mechanisms of action in a single molecule must also be considered, and their role in combination analgesia has yet to be fully determined.

The goal of combination therapy is to achieve analgesic efficacy and safety while reducing the doses of specific single analgesics; in some cases, a synergistic effect may be involved where the end results are greater than the sum of the parts [3]. In addition, combination therapy may delay the start of drug resistance [3]. The aim of this narrative review is to describe combination analgesic regimens, their potential benefits, and the challenges they pose to clinicians.

## Review

Terminology

Multimodal analgesia was developed in the early 1990s by anesthetists and surgeons to achieve more efficacious and safer analgesia with the use of smaller doses of drugs [4]. Multimodal analgesia became an important concept in pain care soon afterward [5], to the point that today 93% of chronic pain patients take more than one analgesic agent; incredibly, in a survey of 3000 chronic pain patients, 176 different drug combinations were reported [6].

Combinations refer to drugs taken concurrently for their additive or possibly synergistic benefits. By definition, a fixed-dose combination product is two or more agents contained in a single dosage form, such as one capsule or one tablet [7]. Sometimes called fixed-ratio products [8], they differ from fixed-dose protocols in which two or more agents are prescribed, but are dosed individually and possibly at different times. For instance, a combination regimen may include one agent requiring weight-based dosing that would necessitate the use of two individual agents albeit used together [9].

A unit dose consists of a prepackaged dose, such as one or more tablets contained in a single blister in a blister pack. For example, a blister pack that consisted of 325 mg acetaminophen and 5 mg oxycodone in two pills housed in one single blister would be a unit dose. On the other hand, one table of 10 mg oxycodone in a blister pack would likewise count as one unit dose.

In some cases, two agents are provided in one pill, such as oxycodone and acetaminophen; these are sometimes called “fixed-dose combination products.” There are a large number of fixed-dose combination products for analgesia, which combine two or more agents in one tablet or capsule at specific doses. It may be more accurate to think of them as coformulations, defined as the provision of more than one drug into one pill, but the use of this term has been extended to include injectables [8]. In a coformulation, both of the agents retain their own pharmacokinetic and pharmacodynamic profiles [8]. Fixed-dose combinations are used to treat multimechanistic forms of pain [10].

The safety and efficacy of fixed-dose combination products

Diclofenac and Thiocolchicoside

In a double-blind, parallel-group study at two centers, patients with acute low back pain were treated with an intramuscular (IM) injection of 4 mL diclofenac plus 75 mg of thiocolchicoside fixed-dose combination in a single injection or the two agents were administered as two separate injections. Patients received an IM injection(s) every day for five days. Both groups had significant pain relief over baseline at five days and there was no significant difference between the groups for pain [11]. This suggests that a single injection of the fixed-dose combination injection was as effective as two separate injections, one of diclofenac and the other of thiocolchicoside.

Drotaverine Hydrochloride and Acetaminophen

A randomized, double-blind, controlled trial evaluated the use of a fixed-dose combination of the antispasmodic, drotaverine hydrochloride, 80 mg, and acetaminophen 500 mg to control abdominal pain associated with acute infectious gastroenteritis. The study compared this combination to acetaminophen 500 mg monotherapy. Patients were dosed three times a day for three days. Combination patients reported significantly better pain control at 2 hours and a more rapid onset of pain control than the monotherapy group [12].

Glucosamine Sulfate and Chondroitin Sulfate

Patients with knee osteoarthritis were enrolled in this randomized, double-blind study to receive either a fixed-dose combination of glucosamine sulfate and chondroitin sulfate for 12 weeks or Cosamin DS® as the comparator drug. Note that Cosamin DS is an over-the-counter fixed-dose product that combines glucosamine 1500 mg with chondroitin sulfate 1200 mg. Both groups exhibited significant pain relief over baseline but there was no significant difference between the groups [13].

Hydrocodone and Acetaminophen

A posthoc data analysis from two companion 12-week trials was reported on patients who had previously been taking a fixed-dose combination product of hydrocodone plus acetaminophen for chronic pain. This was a year-long, open-label study of hydrocodone monotherapy using flexible dosing. At baseline, pain scores averaged 7.3 and of the 57% of patients who completed the 52-week study, mean pain scores were 3.6 to 4.1 during the maintenance phase [14]. This was not a comparative study but showed that patients taking the fixed-dose combination product could be rotated to the single-entity product with good results.

A novel 12-hour drug formulation of biphasic hydrocodone and acetaminophen was evaluated in an open-label study. Each fixed-dose combination tablet contained 7.5 mg hydrocodone (total) and 325 mg acetaminophen. The biphasic hydrocodone comprises both an immediate-release and an extended-release formulation, such that 25% of the hydrocodone was administered as immediate release, followed by the release of acetaminophen, and then concluding with the extended-release of the remaining hydrocodone. Patients with moderate to severe osteoarthritis pain were enrolled and took three tablets as the starting dose and then two tablets daily over the course of 35 days. About a quarter of patients (24.2%) dropped out because of adverse events, which were reported in 57.5% of all patients. The most frequently reported adverse events were nausea, somnolence, and constipation. Thirteen patients had abnormal liver function test results but did not have acute liver failure. Thus, the safety profile of this product was similar to other opioid products, but its use in chronic pain patients must be subject to further study [15].

In a head-to-head study, hydrocodone/acetaminophen was compared to the buprenorphine transdermal patch for patients with osteoarthritis pain. Both products were considered similar in terms of analgesic efficacy and tolerability. Patients took varying doses but they ranged from 15 to 30 mg of hydrocodone per day and buprenorphine patches were 10 or 20 μg/h [16].

Ibuprofen and Acetaminophen

In a single-dose, open-label, five-period crossover, pharmacokinetic study, an intravenous (IV) product consisting of 3 mg/mL ibuprofen and 10 mg/mL acetaminophen delivered concomitantly in a fixed-dose product did not alter the pharmacokinetics of either drug [17].

In a randomized, double-blind, placebo-controlled trial of dental pain, a fixed dose product of ibuprofen 292.5 mg and acetaminophen 975 mg was compared to ibuprofen 292.5 mg alone, acetaminophen 975 mg alone, or placebo for pain control over 28 hours. The fixed-dose combination provided significantly superior pain control and a more rapid onset of action compared to both monotherapies and placebo [18].

A 12-hour dose-ranging, proof-of-concept study among dental surgery patients compared ibuprofen and acetaminophen fixed-dose combination products of 200/500 mg, 250/500 mg, and 300/500 mg, respectively with ibuprofen 400 mg as a comparator and placebo. All of the treatments were significantly superior to placebo but not significantly superior to ibuprofen 400 mg monotherapy. The duration of pain relief was similar with all active therapies (9.7 to 11.1 hours compared to 1.6 hours for placebo) [19].

A randomized, double-blind, multicenter study of bunionectomy patients compared the use of oral ibuprofen 300 mg plus acetaminophen 1000 mg to ibuprofen 300 mg alone and acetaminophen 1000 mg alone, or to a placebo. The study lasted 48 hours and found the combination product offered significantly better analgesia than either monotherapy or the placebo (p<0.001 for all). Safety was similar in all three groups [20].

A randomized, double-blind, parallel-group study of 892 community-dwelling patients with knee pain compared short-term (10 days) and long-term (13 weeks) results of patients taking one of four regimens: a fixed-dose combination tablet of ibuprofen/acetaminophen 200 mg/500 mg; two such fixed-dose tablets taken together (ibuprofen/acetaminophen 400 mg/1000 mg); ibuprofen 400 mg alone; or acetaminophen 1000 mg alone. Patients received doses three times daily. At day 10, two combination pills, taken three times a day, provided significantly better pain control than acetaminophen monotherapy (p<0.01). At week 13, combination-pill patients (either the one-pill or two-pill groups) were more likely than other patients to rate their treatment as “good” or “excellent.” Adverse events were similar in all groups, but at week 13, there were patients in all of the groups who had decreased hemoglobin levels (≥ 1 g/dL), and this decrease occurred in significantly more patients taking the two fixed-dose combination pills twice daily compared to patients on monotherapy. The benefits of combination therapy in this patient population at 13 weeks compared to the other regimens were modest [21].

Ibuprofen and Caffeine

In a placebo-controlled, double-blind, parallel-group study over 8 hours for patients with dental pain, patients were randomized to receive ibuprofen/caffeine 400 mg/100 mg or ibuprofen 400 mg monotherapy or placebo. The combination product was significantly superior in providing pain control over 8 hours than ibuprofen alone or placebo; the ibuprofen/caffeine product provided pain relief significantly faster than ibuprofen alone (1.13 hours versus 1.78 hours, p=0.0001) [22]. A post hoc subgroup analysis of this study found that a better analgesic effect was most pronounced in those patients who had moderate pain levels at baseline [23].

Naproxen and Esomeprazole

A combination oral tablet of enteric-coated naproxen 500 mg combined with 20 mg of immediate-release esomeprazole magnesium was compared to celecoxib 200 mg monotherapy for knee osteoarthritis. While esomeprazole is not an analgesic, patients in the combination group had superior pain relief compared to celecoxib (p<0.05) over 12 weeks [24].

In a six-month observational study, 45 patients with juvenile idiopathic arthritis (ages 12 to 16 years) were treated with naproxen and esomeprazole at least once. The combination product was well tolerated with a high response to the American College of Rheumatology (ACR) criteria [25].

Naproxen, Acetaminophen, and Pamabrom

In a double-blind, single-center randomized trial of women with primary dysmenorrhea >17 years, two combination products were compared. The first was acetaminophen plus pyrilamine, and the diuretic pamabrom (widely used in Mexico, where this study was conducted). This combination regimen was compared to naproxen, acetaminophen, and pamabrom for one menstrual cycle. Both treatments were well tolerated by the women and both relieved pain, but with no significant difference between them [26].

Naproxen and Sumatriptan

Two trials were conducted among adults with dysmenorrhea and menstrual migraines. They were to take a fixed-dose combination product of naproxen/sumatriptan 500 mg/85 mg in a single fixed-dose combination oral product, or a placebo within one hour of the onset of the headache. The primary endpoint was 2 hours of pain-free response. In both studies, the fixed-dose combination product was significantly better than the placebo in pain-free response, and this response lasted up to 48 hours. No serious adverse events were reported [27].

In a study of migraine patients, patients were randomized to one of four groups: a fixed-dose combination group (naproxen/sumatriptan 500 mg/85 mg); sumatriptan 85 mg monotherapy; naproxen 500 mg monotherapy; or placebo. At two hours post-dose, in one of the two studies, fewer patients in the combination group had nausea (71% v. 65%, p=0.007). For sustained "pain-free response" at 2 to 24 hours after the dose, the combination group was significantly larger than both placebo and the monotherapy groups. Adverse events were similar for the fixed-dose combination product and sumatriptan alone [28].

A systematic review and meta-analysis of the use of naproxen combined with sumatriptan for migraines found 13 studies of naproxen/sumatriptan 500 mg/50 or 85 mg and found that combination products were effective for acute pain control with migraine headaches with greater effect than either drug used alone. Adverse events for the combination product were similar to those for sumatriptan monotherapy [29].

Oxycodone and Acetaminophen

In an open-label extension of randomized, double-blind trial patients who underwent a bunionectomy were randomized to acetaminophen/extended-release oxycodone 650 mg/15 mg every 12 hours or a biphasic fixed-dose combination product of oxycodone and acetaminophen, where the oxycodone was offered in both immediate-release and extended-release formulations in the same product (likewise 650 mg acetaminophen and 15 mg oxycodone). Patients were administered these drugs every 12 hours over 14 days. Both products demonstrated good pain control with side effects consistent with opioid products, which occurred in 43.8% of patients [30].

Acetaminophen toxicity is an important consideration, despite the ubiquity of this agent and its prevalence in pain-control regimens. Acetaminophen poisoning is the most important cause of liver transplantation in the United States and the second most frequent around the world [31,32]. In 2010, the U.S. Food and Drug Administration (FDA) recommended reducing the maximum daily dose of acetaminophen from 3900 to 4000 mg per day to 3000 to 3250 mg per day, which initially resulted in confusion as generic manufacturers did not adjust their labeling (since the change was a recommendation rather than a requirement) while branded products did [33,34].

Tramadol and Acetaminophen

A placebo-controlled study of patients with chronic low back pain evaluated extended-release tramadol 75 mg combined with acetaminophen 650 mg in a fixed-dose combination tablet. Although it was mainly a safety study, it also reported that significantly more combination-product patients reported a reduction of ≥30% in pain intensity compared to placebo patients, although combination patients had a significantly higher rate of treatment-emergent adverse events than placebo patients. The most frequently reported adverse events were nausea, dizziness, constipation, and vomiting [35].

Tramadol and Dexketoprofen

A fixed-dose combination product of dexketoprofen 25 mg and tramadol 75 mg was compared in a double-blind study of hip arthroplasty patients to dexketoprofen 25 mg or tramadol 100 mg, taken every 8 hours over a five-day course. The fixed-dose combination product provided superior pain control compared to the single entitles, even at higher doses. Safety results were similar to other opioids [36].

A randomized, double-blind, placebo-controlled trial of dental pain compared a combination of oral tramadol 75 mg and dexketoprofen 25 mg to tramadol 75 mg and acetaminophen 650 mg [37]. Patients were asked to rate the pain at various time points (15, 30, 60, 90, 120, 240, 360, and 480 minutes after surgery) on a 0 to 4 pain scale, where 4 was complete pain relief. Using a double-stop-watch technique, the time to onset of analgesia was also recorded. The tramadol/dexketoprofen regimen was superior to the placebo and the tramadol/acetaminophen combination at all points with similar adverse events [37].

Loose-dose combination analgesia

Although “loose-dose combination analgesia” lacks a consensus definition or any sort of formal definition, it is understood by the authors to involve the use of two or more products dosed separately but used concurrently, in this case, for pain control. The same drug combinations can be used for fixed-dose and loose-dose combinations, but there are reasons to prefer one over the other in certain situations. See Table 1.

**Table 1:** Fixed-dose compared to loose-dose combination analgesic regimens FDA: Food and Drug Administration; PD: pharmacodynamics; PK: pharmacokinetics

	Fixed-Dose Product	Loose-Dose Combination	Comment
Synergistic drug combination	Yes	Depends	A good knowledge of synergistic combinations is required for effective loose dosing
Ceiling effect	Yes	No	
Predictable PK/PD	Yes	No	
For oral products	Reduces pill burden	Does not reduce pill burden	Reduced pill burden may improve compliance
Cleared to market by FDA	Yes	No	Loose dosing may offer clinicians versatility to meet specific individual patient needs
Known adverse events	Yes	Not necessarily	Drugs cleared to market publish safety data but loose dosing may exacerbate side effects in some cases
Adjusted to meet specific needs of individual patient	No	Yes	A primary benefit of loose dosing

Isobolography

Additive effects allow the effects of two or more individual agents to be combined in such a way that neither augments nor decreases the sum of the parts. Synergy has been described as a combination in which the benefits or adverse effects are greater than the sum of the parts. Thus, synergy is more than the additive effect but the actual definition of synergy remains a subject of contention [38]. Without a consensus on how to define, much less how to calculate synergy, the use of this terminology is limited.

There is an arithmetic model for calculating drug synergy (adding the effects of Drug 1 to Drug 2), but this does help to define synergy. For instance, if Drug 1 reduces pain by 50% and Drug 2 reduces pain by 75%, taking them together cannot reduce pain by 125% (50% + 75%). The combination index, a metric for the extent to which a drug interacts at a given level [39], is often used to calculate drug-drug interactions and can be helpful for studying synergy; combination indices are more helpful in this regard than p-values [38]. Further, the dose-effect curve has proven useful for studying synergy, which is influenced by drug potency. Animal studies and clinical trials have been used to support the notion of combination synergy, but synergy is not necessarily equivalent to noninferiority or superiority, which are the common endpoints for such studies. Nor can an in-depth understanding of pain mechanisms help predict synergy because synergy is governed by mass-action law and not mechanism. In other words, the knowledge of the distinct mechanisms of two drugs might grant an intuitive understanding of how they might work together and even be sufficient for formulating a hypothesis, but a mechanism-based understanding of two or more drugs is not quantitative. This gets even more complex with novel molecules with multiple mechanisms of action [38].

Synergy should not be confused with “enhancement,” which is unilateral; synergy must be mutual. For instance, one agent may potentiate another and that can generally be calculated as a percentage potentiation. Synergy, on the other hand, must be calculated using the combination index [38]. Drugs must have mutual effects on each other to be synergistic.

**Figure 1:**
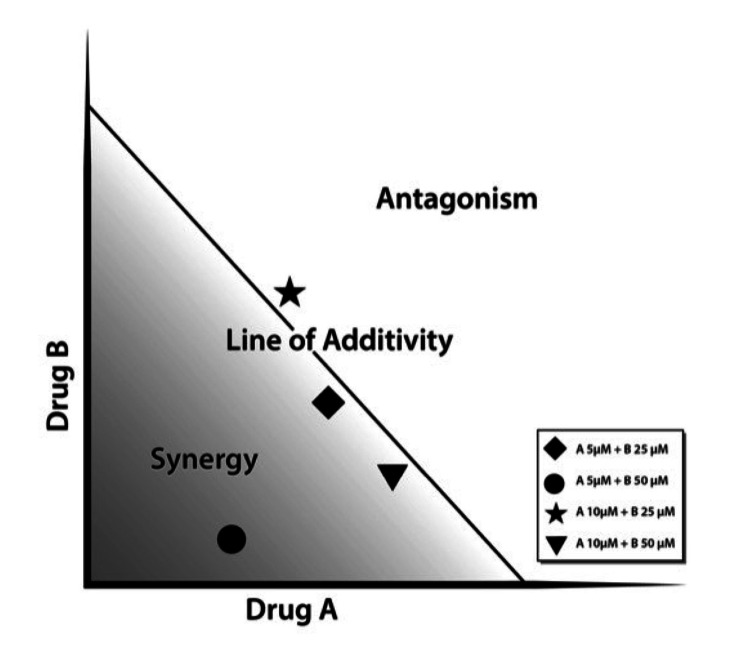
Normalized isobologram for hypothetical drugs A and B This is a normalized isobologram for hypothetical drugs A and B. Note that two drugs may be synergistic at certain concentrations but not others. One coordinate represents the inhibitory concentration (IC) of Drug A, and the other Drug B is required to produce a defined effect, for instance, IC50, A and IC50, B when x=50%. These points, IC50, A, 0 and 0, IC50, B are used to define the Line of Additivity in a 50% effect isobologram. Then concentrations of the drugs that provide the same effect, that is, x=50%, are denoted by other points on the plot.

Known since the late 19th century, the isobologram allows the interaction of two agents at a given effect level to be graphically depicted and thus analyzed [40]. In simplest terms, the concentration of the drug necessary to produce the desired effect (IC50) is determined for both drugs and plotted on the x and y axes of a two-coordinate plot. Thus, synergy, additivity, and antagonism can be plotted in an isobologram [41]. A normalized isobologram for fictional drugs A and B appears in Figure 1.

Important work by Chou and colleagues has led to a computerized algorithm for the Chou-Talay method, which analyzes the dose effect of two or three drugs, allowing for the quantitation of synergism (combination index <1) and antagonism (combination index >1). This algorithm allows precise calculation of the optimal combination and schedule of treatment [3,42]. In fact, computer algorithms have helped facilitate optimal synergistic drug combinations quickly and reliably. Moreover, computer algorithms have vastly changed drug development, allowing researchers to create drugs based on desired outcomes rather than relying on the serendipitous “discovery” of drugs.

Certain drug combinations are known to work synergistically, such as oxycodone and acetaminophen. Other drug combinations may offer only additive benefits or no benefits at all. Combinations also have the potential to exacerbate side effects. It has been suggested that cannabinoids and opioids may offer synergistic analgesic benefits, but no such fixed-dose combination products have come to market [43]. This may be even more complex than a mere synergistic drug combination, in that opioids and cannabinoids may have reciprocal interactions as well [44].

Multimechanistic single-entity analgesics

Long recognized as effective pain relievers, opioids produce their analgesic effects by activation of the μ-opioid receptor pathways. Recent analgesic developments have brought to market certain products that are μ-opioid receptor agonists combined in a single molecule with a nonopioid mechanism of action to enhance pain relief. The four analgesic agents that fit into this new category are tramadol, tapentadol, buprenorphine, and cebranopadol, sometimes collectively called “atypical opioids” [45].

Tramadol

Tramadol is a centrally acting analgesic that offers two mechanisms of action in a single entity. On the one hand, it is a μ-opioid receptor agonist, and on the other hand, it inhibits the neuronal uptake of both norepinephrine and serotonin [46]. The benefit of the latter is that it optimizes synaptic levels of neurotransmitters (pain signals) while at the same time activating inhibitory descending pain pathways [47].

Tramadol has an active metabolite (O-destmethyltramadol) which binds to the μ-opioid receptors with greater affinity than the parent molecule [48]. The two mechanisms of action (opioid plus nonopioid) have a synergistic effect [49], and it is hypothesized that tramadol exerts an anti-inflammatory effect as well [50]. Moreover, tramadol can be used in combination with other analgesics, such as fixed-dose combination products of tramadol and acetaminophen for chronic non-cancer pain [51,52].

Tapentadol

Tapentadol is a novel first-in-class agent that is often grouped with opioids, although it is clearly an atypical opioid [53]. Tapentadol provides both μ-opioid receptor agonism and also inhibits the neuronal reuptake of norepinephrine and serotonin, making it similar to tramadol, but unlike tramadol, tapentadol has no active metabolites and is metabolized by glucuronidation (phase 2 metabolism) which may reduce the risk of drug-drug interactions [53]. Although tapentadol is less toxic than conventional opioids, adverse effects such as potentially life-threatening respiratory depression are possible [54]. Tapentadol is not likely to induce serotonin syndrome [54].

Tapentadol has been shown to be effective in treating neuropathic pain conditions, including chemotherapy-induced peripheral neuropathy [55]. Tapentadol has also been shown to be an effective analgesic agent for low back pain [56]. Because tapentadol has less activity at the μ-opioid receptors compared to conventional opioids such as morphine or oxycodone, it has fewer opioid-associated adverse effects [57].

Cebranopadol

Cebranopadol is the newest of these four agents and it combined dual agonist action at the opioid and the nociception/orphanin (NOP) peptide receptors [46]. It is a first-in-class novel drug with a central mechanism of action. As such, it offers antinociception combined with an anti-hyperalgesic effect for use in both acute and chronic pain conditions. In contrast to other opioids, it is generally more effective against neuropathic than nociceptive pain [46].

Sometimes called the ORL-1, the nociception FQ peptide (NOP) receptor works by mediating the effects of the endogenous peptide ligand nociception/orphanin FQ (N/OFQ) [58,59]. N/OFQ has little to no affinity for the main opioid receptors; in fact, the NOP receptor is relatively insensitive to morphine and does not respond to naloxone. NOP receptors are located throughout the central nervous system.

Cebranopadol combines NOP receptor agonism with μ-receptor agonism which appears to dampen the opioid reward pathways in a manner reminiscent of buprenorphine [60,61].

In a nonrandomized, open-label study of cancer pain patients, cebranopadol at doses ranging from 200 to 1000 μg/d was used over 26 weeks and found that it offered safe, well-tolerated pain control [62]. In a murine study, it was shown that the activation of both NOP and opioid receptors helped to counteract the tendency toward physical dependence on the drug [63].

Buprenorphine

Buprenorphine is often described as a unique agent as it has attributes that set it apart from most other opioid analgesics [64]. Buprenorphine is a partial agonist with high binding affinity at the μ-opioid receptors but it acts as an antagonist with high binding affinity at the δ- and κ-opioid receptors. Buprenorphine is also an agonist of the opioid-receptor-like 1 (ORL1) receptor with low binding affinity [65]. It is a misconception that because buprenorphine is a partial agonist at the μ-opioid receptors that it provides only a partial analgesic effect [66]. From an analgesic viewpoint, buprenorphine provides full analgesic benefits at the μ-opioid receptors [66]. This chemical structure may allow buprenorphine to mediate signaling from the spinal opioid receptors more than other conventional opioids, controlling pain, with less effect on brain opioid receptors, limiting euphoria and the risk of opioid use disorder.

Co-crystals

The field of crystal engineering has allowed for supramolecular synthesis in which existing agents can be combined into new forms without the need to break covalent bonds [67]. Co-crystals have been combined with nutraceuticals to address chronic pain symptoms as have other combinations, such as ketoprofen and gabapentin [68]. Acetaminophen and caffeine represent a frequently used over-the-counter drug combination that is being evaluated as a co-crystal combination which may improve its pharmacological properties to the extent that a reduced dose of acetaminophen could be used for equianalgesic effect [69]. A novel co-crystal combination of tramadol plus celecoxib is current in phase 3 clinical trials. This co-crystal formulation may allow for slower tramadol absorption with faster celecoxib absorption compared to the loose doses of these two reference agents [70]. Co-crystalization may help optimize the characteristics of drug combinations, such as their stability, bioavailability, and solubility [71-74].

## Conclusions

The use of combination analgesics with multiple and complementary mechanisms of action is well-founded in pharmacologic science. Pain can be multimechanistic and combination analgesia with agents having different mechanisms of action may better address complex forms of pain compared to pharmacologic therapy relying solely on one mechanism of action. Studies of certain combination products show that they may, in some cases, offer greater analgesic benefits and/or fewer side effects.
